# The CD112R/CD112 axis: a breakthrough in cancer immunotherapy

**DOI:** 10.1186/s13046-021-02053-y

**Published:** 2021-09-10

**Authors:** Taofei Zeng, Yuqing Cao, Tianqiang Jin, Yu Tian, Chaoliu Dai, Feng Xu

**Affiliations:** Department of General Surgery, Shengjing Hospital of China Medical University, 36 Sanhao Street, Heping District, Shenyang, 110004 China

**Keywords:** CD112R, PVRIG, CD112, Immune checkpoints, Cancer immunotherapy, Cancer prognosis

## Abstract

The recent discovery of immune checkpoint inhibitors is a significant milestone in cancer immunotherapy research. However, some patients with primary or adaptive drug resistance might not benefit from the overall therapeutic potential of immunotherapy in oncology. Thus, it is becoming increasingly critical for oncologists to explore the availability of new immune checkpoint inhibitors. An emerging co-inhibitory receptor, CD112R (also called PVRIG), is most commonly expressed on natural killer (NK) and T cells. It binds to its ligand (CD112 or PVRL2/nectin-2) and inhibits the strength with which T cells and NK cells respond to cancer. Therefore, CD112R is being presented as a new immune checkpoint inhibitor with high potential in cancer immunotherapy. CD112 is easily detectable on antigen-presenting or tumor cells, and its high level of expression has been linked with tumor progression and poor outcomes in most cancer patients. This review explores the molecular and functional relationship between CD112R, TIGIT, CD96, and CD226 in T cell responses. In addition, this review comprehensively discusses the recent developments of CD112R/CD112 immune checkpoints in cancer immunotherapy and prognosis.

## Background

Immune checkpoint inhibitors (ICIs)’ potential to treat certain types of cancer has been documented recently, with encouraging results [1–5]. The FDA’s approval of Ipilimumab, an antibody targeting cytotoxic T lymphocyte-associated antigen-4 (CTLA-4), is considered a milestone in cancer treatment and has led to substantial patient outcomes unresectable or metastatic melanoma [6–9]. Subsequently, pembrolizumab and nivolumab, antibodies that block programmed cell death protein-1 (PD-1), were effectively used to treat a variety of tumors, such as renal cell carcinoma (RCC) [10], advanced melanoma [10, 11], non-small-cell lung cancer (NSCLC) [10], breast cancer [5, 12], and advanced hepatocellular carcinoma [13]. Furthermore, the anti-PD-1 drugs demonstrated better survival rates than conventional therapies when used for cancer immunotherapy [4, 5].

However, a substantial number of cancer patients either failed to respond to these single-agent immune checkpoint inhibition therapeutics or demonstrated an initial response followed by acquired resistance [14–18]. Effector T cells exhibit the exhausted phenotype and malfunction within the tumor microenvironment (TME) [19–23]. Dysfunctional T cells have increased numbers of co-inhibitory receptors, such as CTLA-4 and PD-1, on their surfaces. Based on these facts, identifying new immune checkpoint pathways might improve the immunotherapy response rates and broaden the efficacy of the treatment provided. New therapies that target other co-inhibitory receptor pathways belonging to the nectin and related families (such as CD96, T cell immunoglobulin and ITIM domain (TIGIT), and CD112R) have been validated in cancer immunotherapy [24–26]. The present study highlights the unique characteristics of the CD112R-CD112 co-inhibitory signal pathway and discusses how it modulates antitumor immunity.

### Structure and expression of CD112R

#### Structure of CD112R

**Fig. 1:**
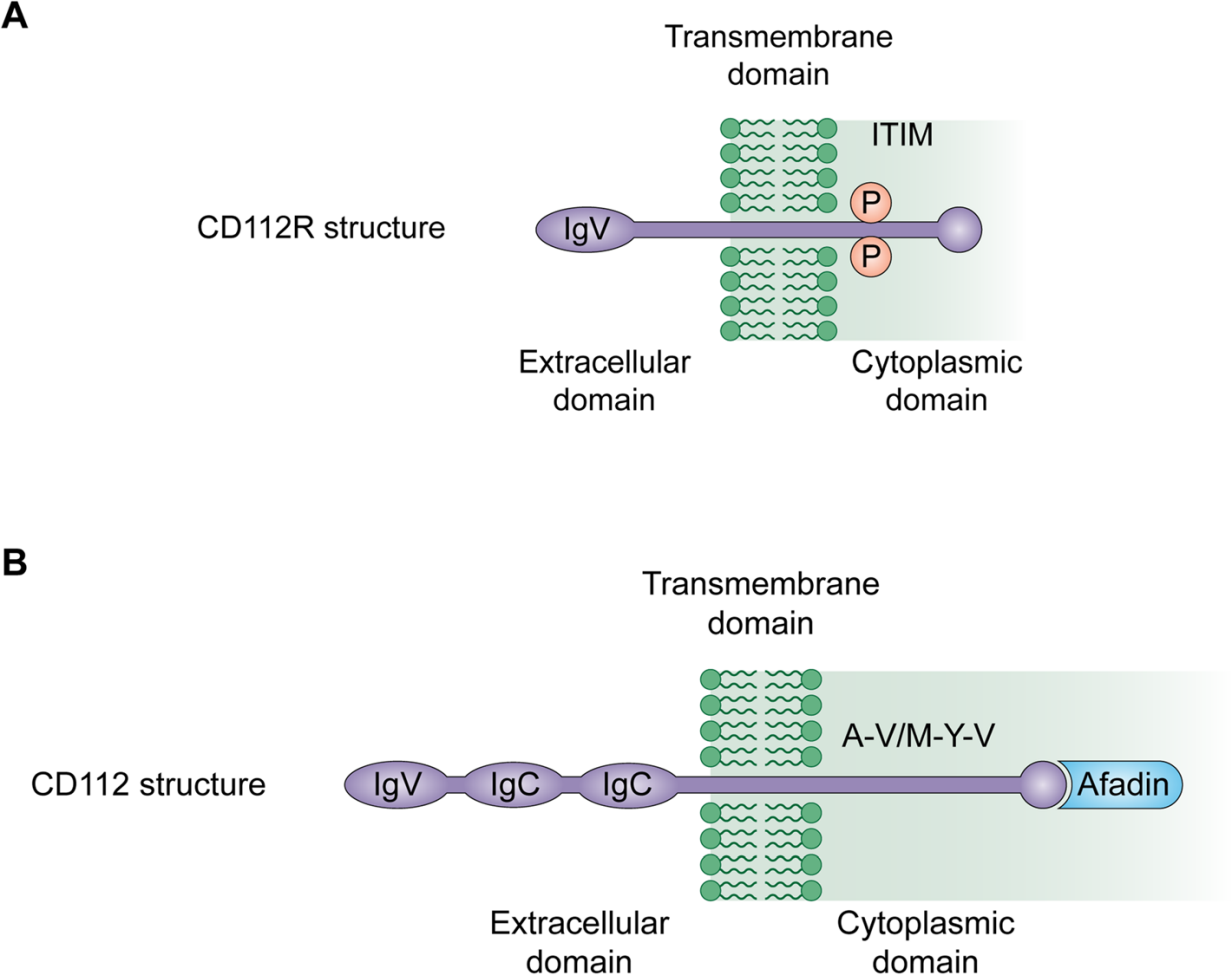
CD112R and CD112 structures. **A**. CD112R protein includes a single extracellular IgV domain, one transmembrane domain, and a long intracellular domain with an ITIM-like motif. **B**. CD112 protein is composed of an extracellular region with three Ig-like domains (a distal IgV domain and two IgC domains), a single transmembrane region, and a cytoplasmic tail possessed a conserved afadin-binding motif

Initially named and described by Zhu et al. in 2016, CD112R is an emerging co-inhibitory receptor that belongs to the poliovirus receptor (PVR) family [27]. The gene was initially referred to as a PVR-related Ig domain (NCBI nucleotide database under the number BC073861) and goes by the name PVRIG. In humans, a 36kD single-pass transmembrane protein (Fig. 1A) that includes one transmembrane spanning region, a long intracellular domain, and a single extracellular immunoglobulin variable-like (IgV) domain is encoded inside the CD112R. In humans, the CD112R gene consists of a tail sequence with ∼65.3% similarity to that of mice [27]. The single extracellular IgV domain conserves three motifs from the PVR family: Tyr139 or Phe139-Pro140-X-Gly142, Ala89-X6-Gly96, and Val, Ile-Ser, and Thr-Gln at position 72–74 AA of CD112R [27, 28]. The intracellular domain consists of two tyrosine residues: Y233 and Y293. Y233 is inside an ITIM-like motif linked to phosphorylation sites [29]. The phosphorylation of Y233 played a significant role in CD112R-mediated signal transduction, but Y293’s effects were negligible [27].

#### Expression of CD112R in healthy conditions

**Fig. 2:**
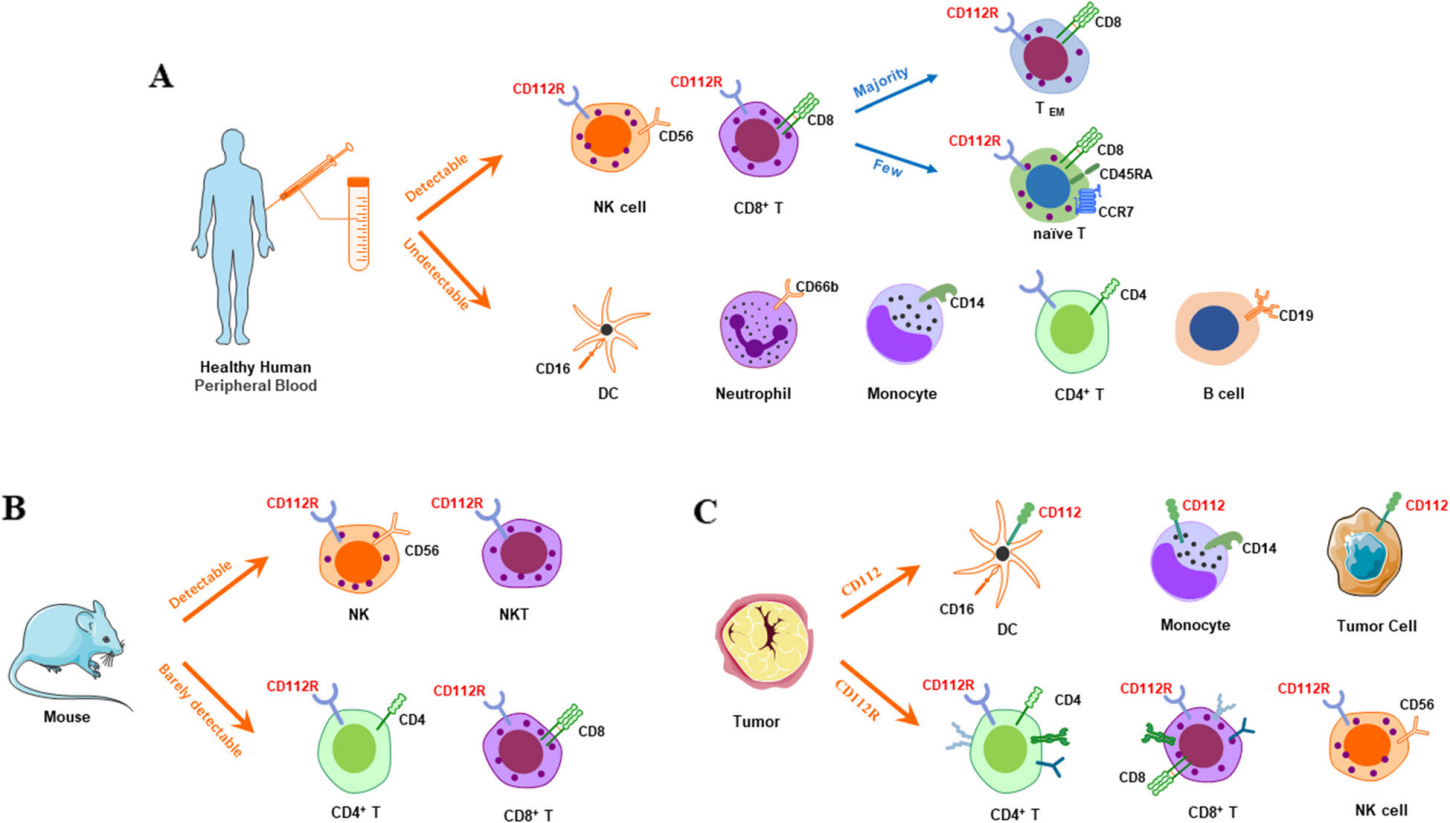
CD112R and CD112 expression in health and tumor microenvironment. (**A**) In normal human peripheral blood cell subsets, CD112R is predominantly expressed in NK cells and CD8^+^ T cells, but not in CD4^+^ T cells, DCs, neutrophils, monocytes and B cells. CD112R^+^CD8^+^ T cells are mainly detected in effector memory T (CD45RA^−^CCR7^−^) cell in phenotype, and rarely in naïve T (CD45RA^+^CCR7^+^) cells. (**B**) In mouse, CD112R are detectable in NK cells and NKT cells, but are barely detectable in CD4^+^ T cells and CD8^+^T cells. (**C**) In the tumor microenvironment, CD112R is expressed abundantly in NK cells, CD4^+^ T cells and CD8^+^ T cells. Meanwhile, CD112 was detectable primarily in DCs, tumor-associated macrophages (TAMs) or monocytes, and tumors cells

In normal human peripheral blood cell subsets (Fig. 2A), the CD112R gene is expressed in NK and T lymphocyte cells [27, 30], but not in monocyte-derived dendritic cells (DCs) [27], neutrophils (CD66b^+^), monocytes (CD14^+^), and B cells (CD19^+^). Most T cells that express CD112R are CD8^+^ T cells, which are primarily effector/memory cells [27]. Very few of CD112R positive cells are naïve T cells (CD45RA^+^CCR7^+^) [27]. CD112R is not detected in CD4^+^ T helper cells from peripheral blood [27]. Remarkedly, the activation of both CD8^+^ and CD4^+^ T cells further upregulates CD112R gene and protein expression [27]. In mouse cells (Fig. 2B), CD112R transcripts are highly expressed on NK cells and NKT cells but are barely detectable in CD4^+^ T cells, CD8^+^ T cells [31, 32]. Unlike human T lymphocyte cells, activated mouse CD4^+^ T cells did not increase CD112R expression, while activated mouse CD8^+^ T cells did [31].

#### CD112R expression in human tumors

CD112R expression has occurred in NK cells, CD8^+^ T cells, and CD4^+^ T cells in various types of solid tumors (Fig. 2C); the highest expression levels appeared in tumors of the kidney, ovary, lung, prostate and endometrium [33, 34]. Likewise, CD112R expression occurred in T cells and NK cells in acute myeloid leukemia, with lower expression levels in the CD8^−^ T cells and higher expression levels in the CD3^+^CD56^+^ NKT, CD3^−^CD56^+^ NK, and CD8^+^ T cells [35].

Exhausted T cells exhibit higher levels of CTLA-4 and PD-1 co-inhibitory receptors on their surfaces [36]. CD112R expression occurred alongside PD-1 and TIGIT expression on CD8^+^ and CD4^+^ TILs, which was significantly correlated with exhausted T cell phenotype [33]. Furthermore, the expression of CD112R also occurred in conjunction with that of exhaustion markers on NK cells, including CD96, TIGIT, Tim-3 and PD-1 [24, 37].

### Structure and expression of CD112

CD112 (cluster of differentiation 112), also known as nectin-2 or PVR-related protein 2 (PVRL2), is a member of the nectin family and has been repotored to correlate with tumor angiogenesis, growth, and metastasis [38, 39]. CD112 shares the same domain with other members of the nectin family (Fig. 1B). It comprises an extracellular region with three Ig-like domains (two IgC and one distal IgV domains), a cytoplasmic tail, and a single transmembrane region. The cytoplasmic tail has a preserved afadin-binding motif (Glu/Ala-X-Tyr-Val), where the postsynaptic density-95, disc large, zonula occludens-1 domain (PDZ domain) of afadin is bound and thus helping the nectins to connect to F-actin [40].

Previous studies have shown that CD112 is mainly localized at the adherent junction in epithelial cells and ubiquitously expressed in various cell including epithelial cells, endothelial cells, neurons, and fibroblasts [38, 41–43]. Furthermore, CD112 also appears in cancer cells and immune cells (Fig. 2C). Immunofluorescence and FACS analysis on a panel of tumor cell lines revealed that CD112 was mainly expressed in the intracellular compartment [43]. High levels of CD112 occurred on human DCs derived from monocytes, and this expression was further up-regulated with the addition of Toll-like receptors (TLR) agonists [27]. Recently, Sarah Whelan et al. found that CD112 was highly expressed on CD14^+^ cells (probably tumor-associated macrophages [TAMs] and monocytes) and CD45^−^ cells (such as tumor epithelial cells and other nonimmune system cells) in breast, endometrial, ovarian, lung cancer, and liver cancer [33, 44]. The expression levels were often concomitant with CD112R in the TILs [27, 33], whereas most of the tumor cells from the hematopoietic system did not express CD112 [27]. The expression of CD112R on CD8^+^ T cells and CD112 on TAMs and CD45^−^ cells were detected in the same tumor sample, indicating that the CD112R-CD112 pathway could be co-expressed in the same cancer [33].

### CD112/CD155 and CD112R/TIGIT/CD96/CD226 pathways in T cells

#### Co-inhibitory network and binding affinities

A complicated signaling network regulates T cell activation. Among the co-inhibitory pathways, members of the PVR and PVR-like family, including CD112R, TIGIT, CD96 (also known as T cell activation, increased late expression of TACTILE), CD155 (or PVR, TAGE4, NECL-5) and CD112, are under intense investigation [24].

**Fig. 3:**
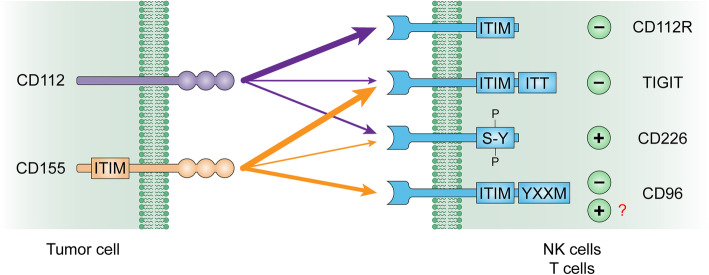
CD112/CD155 and CD226/TIGIT/CD112R/CD96 pathway: CD226, TIGIT, CD112R, and CD96 on T and NK cells bound with their ligands CD112 and CD155 on tumor cells with different affinity. Upon binding, residues in cytoplasmic tails of CD226, CD112R, TIGIT, and CD96 were phosphorylated and produced co-stimulatory or co-inhibitory signals. (reviewed elsewhere [55])

CD112 and CD155 were initially suspected of facilitating the herpes simplex virus and poliovirus spread from one cell to another because they belong to the nectin and nectin-like family. Using heterophilic or homophilic interactions, CD112 and CD155 can initiate intracellular signal transduction, enable tissue organization, and play a role in cell polarity [45]. The ligands can regulate the activity of lymphocytes by binding to different receptors; CD155 interacts with CD226, TIGIT, and CD96, while CD112 binds to CD226, TIGIT, and CD112R (Fig. 3). In spite of this, they do so with strikingly different affinities (Table 1).

**Table 1:** Ligand binding affinities for human CD112R, TIGIT, CD226, and CD96

Ligand	Affinity nMol/L			
	CD112R	TIGIT	CD226	CD96
**CD155**	–	1–3	114–199	37.6
**CD112**	88	Binding fragile	8970 or 310	No binding

CD112R was identified as a new co-inhibitory receptor that inhibited the nuclear factor of activated T cells (NFAT), which mediates T cell activation and is controlled by co-stimulatory signals [27, 46]. No PVR-related protein interacted with the CD112R gene in humans, except CD112 [27]. The affinity of CD112R for CD112 is higher (Kd, 88 nmol/L) than that between CD226 and CD112 (Kd, 8.97 μM or Kd, 0.31 μM) as determined by the Biacore experiments [27, 47, 48]; conversely, the affinity of TIGIT for CD112 is so weak (6 mmol/L) [49] that the interaction can hardly be observed via surface plasmon resonance or ELISA [27, 33]. The CD112R-CD112 interaction was also conserved in mice [27, 31]. Murter et al. generated CD112R-deficient mice and confirmed that murine CD112R is an inhibitory TCR that undermines the antigen-specific activities of CD8^+^ T cells in a CD112-dependent manner [31]. Furthermore, the affinity of mouse CD112R for mouse CD155 was ten times lower than that of mouse CD112R for mouse CD112 [31].

TIGIT, a co-inhibitory receptor that belongs to the immunoglobulin (Ig) superfamily, has a high affinity toward CD155 (Kd, 1–3 nmol/L) and a weaker affinity toward CD112. Although the binding affinity between TIGIT and CD112 is lower than that between TIGIT and CD155, the interactions between TIGIT and the two ligands are similar [50] It was initially discovered in 2009 by different researchers [28, 51, 52]. It can be detected in memory and activated T cells, regulatory T cells, NK and NKT cells, follicular helper T cells, and cytokine-induced killer cells [45]. Studies have shown that TIGIT can dampen the response of the T cell in an extracellular and cell-intrinsic manner through the actions of CD155 and CD112 [28, 53].

CD226 is a co-stimulatory receptor found on NK cells, monocytes, B cells, and T cells [45]. The receptor was initially detected in cytotoxic T cells in response to the lineage-specific activation antigen in humans. The CD226 contains a cytoplasmic tail and two Ig-related extracellular domains [24]. Either CD155 or CD112 can interact with CD226 to co-activate T cells,but CD226 has a higher affinity toward CD155 (Kd, 114–199 nmol/L) than CD112 [45]. In addition, the stimulatory receptor CD226 is outcompeted by the negative receptor TIGIT for CD155 because the affinity between TIGIT and CD155 is markedly higher than that between CD226 and CD155 [45].

Human CD96 selectively binds to CD155 with a higher affinity (Kd, 37.6 nmol/L) than CD226 but lower than TIGIT [27, 28]. CD96 on NK and T cells binds to CD155 and functions as a negative immunoregulatory receptor [54]. However, interactions between CD112 and CD96 have not been reported so far. The CD96 signaling pathway has been mostly studied in NK cells, but the expression of CD96 in human T cells and TILs needs to be evaluated in detail.

#### The regulatory relationship between CD112, TIGIT, CD112R and CD226

Different inhibitory and co-stimulatory checkpoint receptors can co-regulate each other. Co-expression of CD112R with TIGIT was observed in CD8^+^ T cells when the T cells were activated with a specific antigen [33]. CD112R blockade resulted in induction of TIGIT expression, which was not the result of the increased T cell stimulation. No changes in CD112R expression were observed after the blockade of TIGIT or PD-1 [33]. Due to the same binding sites on CD112 shared by CD226 and CD112R [55], CD226 competes with the inhibitory immune checkpoint CD112R binding to CD112 to promote T cell activation [27]. Therefore, CD112R infusion can markedly inhibit the CD112–CD226 interaction in T cells.

In various cancers, TIGIT expression is greatly increased alongside the expression of PD-1 on TILs [56]. Cancer patients tend to present with higher TIGIT and lowered CD226 levels than healthy individuals [50]. In CD8^+^ T cells, TIGIT’s high expression level was negatively linked with that of CD226 [50]. In one study, anti-PD-1 on PD-1^+^Tim-3^+^ tumor-specific CD8^+^ T cells was associated with the up-regulation of TIGIT in melanoma [57]. Although CD226 transcripts in CD112R^−/−^CD8^+^ TILs did not appear to be up-regulated in an MC38 tumor model (colon cancer) [31], several inhibitory receptors, including TIGIT, Tim-3, PD-1, and LAG-3, were highly expressed on CD8^+^ T cells that infiltrated the CD112R^−/−^ mouse tumors compared to the wild-type CD8^+^ T cells [31]. Another study reported that CD112 expression can be enhanced if the TILs are reactivated with anti-CD3 and anti-CD28 [33]. Therefore, competitive or cooperative communications between CD112R and other immune receptors and their related ligands control immune cell infiltration and activation in tumor microenvironment.

### Interaction between CD112 and CD112R as a novel therapeutic

#### Role of CD112-CD112R interaction on T cell function

Early studies showed that CD112 could mediate a co-stimulatory effect on T cell response in a cell-extrinsic manner via CD226, a co-stimulatory receptor for CD112 on T cells [58, 59]. However, recent study have increased our understanding of CD112 as a co-inhibitory ligand. CD112 interacts with CD112R and TIGIT to inhibit T cell proliferation, but the affinity of TIGIT for CD112 is weak (6 mmol/L) [49] and undetectable [33].

Blockade of either CD112R or TIGIT slightly increased cell division and cytokine production in CD4^+^ T cells [27]. The combined CD112R and TIGIT blockade greatly facilitated the diffusion of CD4^+^ T cells and enhanced the secretion of cytokines such as IFN-γ, IL-13, IL-10, IL-5, and IL-2 [27]. Besides inducing CD4^+^ T cell responses, TIGIT and CD112R blockade enhanced the cytotoxic role and expansion of CD8^+^ T cells [27, 33]. Further, anti-CD112R combined with anti-TIGIT has a higher effect on T cell activation than combination of CD112R blockade and PD-1 blockade [33]. Extremely, triple blockade of CD112R, TIGIT, and PD-1 resulted in the most significant increase in IFN-γ to enhance CD8^+^ T cell effector function [33]. A synergistic effect of these three inhibitory receptors appeared in antigen-specific T cell responses [27, 33].

#### Role of the CD112/CD112R Axis in Cancer therapy

In humans, the expression of CD112R has occurred on T cells and NK cells in multiple types of tumors [27, 33]. Both CD112R and CD226 compete with each other to bind to CD112. There is a high tendency for CD112R to interact with CD112, and the CD112R/CD112 axis is known to impede the immune function of T cells. Like the well-understood PD-1/PD-L1 pathway in cancer immunotherapy, the novel CD112R-CD112 pathway, which is still being studied (Table 2), might gain popularity regarding tumor immunotherapy shortly.

**Table 2:** Preclinical experiments and clinical trials in promising cancer target of CD112/CD112R

Tumor types	Treatment	Key results	References
melanoma and colon cancer	CD112R deletion; anti-CD112R blocking Ab, alone or in combination with anti-PD-L1 inhibitors	Increased the CD8^+^ T cells immune cell tumor infiltration; improve the antitumor body defense and suppress refractory tumor progression	[31]
endometrial, ovarian, kidney, head and neck, and lung cancers	single treatment and combination of anti-CD112R, anti-PD-1, and anti-TIGIT	Enhanced CD8^+^ T-cell effector in cancers	[33]
breast cancer	Combination of CD112R and TIGIT mAbs	Boosted cytokine production by NK cells and improved NK cell cytotoxicity and tumor-killing effect	[26]
primary peritoneal carcinoma and microsatellite-stable colorectal cancer	single-agent anti-CD112R (COM701), combination with anti-PD-L1 inhibitor nivolumab	The clinical benefit rate was 69% in the COM701 cohort and 75% in the combination group; Pelvic metastases have regressed notably.	[62, 63]

The CD112R-CD112 pathway plays a vital role in regulating the process by which T cells kill tumor cells. The tumor growth stopped in CD112R-deficient mice models of melanoma and colon cancer [31]. Tumors obtained from CD112R^−/−^ animals exhibited increased tumor immune cell infiltration, particularly the CD8^+^ T cells. Activation of tumor-related antigens such as p15E corresponded with a marked increase in the amount of cytokine secreted by CD8^+^ T cells in CD112R^−/−^ tumors compared to those in the wild-type tumors [31]. Remarkably, PD-L1 blockade suppressed the colon tumor growth in CD112R^−/−^ mice but not in the wild-type mice. Further, anti-CD112R blocking Ab, alone or in combination with PD-L1 inhibitors, was found to improve the antitumor body defense and suppress refractory tumor progression in colon carcinoma models [31].

Anti-CD112R led to an increase in IFN-γ production by purified TILs, similar in potency to anti-TIGIT or anti-PD-1 [33]. The combined blockade of TIGIT and PD-1 had an add-on effect on the infiltration, cytokine production, and secretion of Granzyme B in tumor-specific T cells compared to the blockade of PD-1 alone [57]. Additional increases in IL-2 and IFN-γ levels occurred following treatment with anti-TIGIT and anti-CD112R compared to those who underwent monotherapy for lung cancer. However, the triple combined blockade of PD-1, CD112R, and TIGIT was not associated with the further production of IFN-γ or IL-2 in CD3^+^ TILs [33].

Also, blockade of human CD112R contributes to the anti-tumor efficacy of NK cell and inhibits tumor growth. In vitro, CD112R or TIGIT blockade increased the number of IFN-γ^+^ NK cells in breast cancer cells co-cultured with human NK cells. The combination of CD112R and TIGIT mAbs induced additional increases of IFN-γ-producing NK cell and improved NK cell cytotoxicity. CD112R and TIGIT blockade boosted cytokine production by NK cells and improved the tumor-killing effect of trastuzumab [26]. Recently, Li et al. found that either early or late CD112R blockade were effective in inhibiting tumor growth and prolonging the survival of tumor-bearing mice, which was related to the enhanced frequency and cytotoxic potential of tumor-infiltrating NK cells. They also demonstrated that both NK cell and CD8^+^ T cell played an important role in antitumor efficacy of CD112R blockade, and the antitumor effect remained even in the case of adaptive immune deficiency [37].

CD112 might actively regulate the function of T cells in several types of cancers, such as endometrial, breast, prostate, and ovarian, which presented with the highest ratios of CD112 to CD155 [33]. However, colorectal cancer, melanoma, and esophageal cancer present with higher levels of CD155 compared to those of CD112. Endometrial cancer has the highest number of CD155^+^CD112^+^ tumor cells. Kidney and colorectal cancer samples presented with high proportions of CD155^+^CD112^−^ tumor cells [33]. These findings indicate that the type of tumor determines the relative advantages of the TIGIT-CD155 and CD112R-CD112 pathways, which is important for choosing the most appropriate treatment (single agent or combination).

CD112 expression is particularly high in lung, breast, and ovarian cancers. CD112 expression occurs in both PD-L1^−^ and PD-L1^+^ tumors in breast, ovary, and lung tissues; hence, the CD112R-CD112 axis could stifle T cell function in PD-L1^−^ tumors [33]. These findings indicate the need to develop a new treatment strategy by targeting CD112/CD112R for PD-L1^−^ tumors or cancer patients resistant to other immune checkpoint inhibitors.

The recent discovery of PD-1 and CTLA-4 receptor-ligand interactions indicates that the search for effective immunotherapy interventions in cancer treatment is edging toward a long-lasting solution and better survival rates among cancer patients [60, 61]. Presently, a highly reactive anti-CD112R drug, known as COM701, is in its initial clinical trial phase (NCT03667716). Its safety, tolerability, pharmacokinetics, and preliminary efficacy for treating advanced solid tumors, as a single therapy or combined with an anti-PD-1 drug known as nivolumab, is being determined [62]. This trial is being conducted in patients with breast, ovarian, endometrial, and non-small cell lung cancer, and early signs of antitumor efficacy have been reported [63].

#### Relationship between the expression of CD112R and cancer prognosis

CD112R was expressed in NK cells and T cells in many types of tumors. Nonetheless, only a few studies focused on the effect of CD112R on the prognosis of cancer. In one study, the authors analyzed the immune cell types of hepatocellular carcinoma (HCC) samples from TCGA and GEO databases and the genes involved in the immune cells that affected the disease outcome [64]. They found that the CD112R gene was significantly positively associated with overall survival (OS) and recurrence-free survival (RFS), and had a positive association with other checkpoint molecules (PD-1, CTLA4, LAG3, TIM-3, and PDL-1) and T_eff_ (effector T cell) gene signatures. Qiao et al. studied the expression profiles of 553 HCC patients from TCGA and GEO databases and identified a signature comprising eight genes (DCAF13, FAM163A, GPR18, LRP10, CD112R, S100A9, SGCB, and TNNI3K), which predicted the survival of the patients. According to this gene profile, HCC patients could be divided into high-risk and low-risk groups. The expression levels of GPR18, CD112R, and TNNI3K were up-regulated in patients with low-risk scores, while those of DCAF13, FAM163A, LRP10, SGCB, and S100A9 were down-regulated. Patients with a low-risk score were significantly better than those with a high-risk score (2.20 years vs. 8.93 years) [65]. It has been proved that the immunogenic tumor microenvironment (hot tumor) is composed of a large number of PD-L1 positive T cell infiltration, which is associated with favorable prognosis [66, 67]. As mentioned above, the activation of T cells further upregulates CD112R expression. It is speculated that the favorable prognostic value of CD112R positive tumor-infiltrating immune cells in cancers may be associated with the robust antitumor immunity.

#### CD112 expression as a diagnostic and prognostic biomarker

A growing number of preclinical and clinical studies have underscored the role of CD112 in tumor progression, highlighting its implications for tumor prognosis. CD112 expression was heightened in several cancer types, and its overexpression is associated with different tumor outcomes in different types of tumors (Table 3). It has a robust prognostic impact on the OS and progression-free survival (PFS) in some cancers.

**Table 3:** Studies reporting CD112 as a diagnostic and prognostic biomarker

Reference	First Author	Year	Type of cancer	Sample size	comments
[68]	Miao et al.	2013	Gallbladder cancer	46 SC/ASC^a^ 80 AC^b^	CD112 expression was associated with aggressiveness and poor prognosis
[44, 73]	Daniel et al. Huang et al.	2021 2014	Liver cancer	159	Low CD112 expression was associated with poor OS of patients, but contradicted by evidence from preclinical studies
[80]	Karabulut et al.	2015	Colorectal carcinoma (CRC)	140	Serum CD112 levels have a diagnostic value and high levels correlated with an adverse prognostic impact on PFS patients with early-stage
[83]	Liang et al.	2015	Pancreatic Ductal Adenocarcinomas	106	CD112 expression was associated with the progression and poor prognosis
[84]	Izumi et al.	2015	Pancreatic adenocarcinoma	49	CD112 was not associated with overall survival, but higher CD112 expression correlated with worse histological grade.
[77]	Stamm et al.	2018	AML	429^c^	High CD112 expression correlated with shorter overall survival
[76]	Erturk et al.	2019	Lung cancer	74	Serum CD112 expression level was a reliable diagnostic but not prognostic or predictive biomarker.
[81]	Bekes et al.	2019	Ovarian cancer	60	CD112 expression supported tumor cell adhesion, leading to growth and lymph node metastasis.

^a^SC/ASC: squamous cell/adenosquamous carcinoma

^b^ AC: adenocarcinomas

^c^including 139 AML patients enrolled in the AMLSG 07–04 study of the German-Austrian Study Group (NCT00151242) [78] and 290 AML patients in the GEO database (GEO accession GSE6891) [79]

### Gallbladder cancer

According to findings from a cohort of 46 squamous cell/adenosquamous carcinomas (SC/ASC) of the gallbladder and 80 adenocarcinomas (AC), CD112 can be a proliferative and survival factor [68]. Positive CD112 expression was associated with large tumor size, high TNM stage, and lymph node metastasis in the AC and SC/ASC, suggesting that CD112 plays a role in tumor cell proliferation and apoptosis. The AC and SC/ASCs tumor cells were more likely to invade and metastasize in patients with positive CD112 expression [68]. Cell adhesion loss might be required for tumor cells to acquire their invasive abilities. The expression of adhesion molecules on the surface of tumor cells is necessary to establish metastatic foci at the secondary site [69–71]. Therefore, the role of CD112 in the invasion and metastasis of AC and SC/ASC cannot be fully explained by its role as an adhesion molecule. CD112 expression was significantly associated with poor differentiation in AC, but not SC/ASC. Most importantly, Kaplan-Meier survival analysis in the SC/ASC and AC patients revealed that a high CD112 expression level was a strong and independent predictor of a shorter survival time [68]. Hence, CD112 expression is associated with aggressiveness and a poor prognosis in AC and SC/ASC patients.

### Liver cancer

A recent study showed that CD112 was overexpressed in HCCs compared with that of adjacent tissues based on RNA sequencing (RNA-seq), single cell RNA-seq (scRNA-seq) and IHC, and HCC tumors were significantly smaller through recovering T cell infiltration, and tempering T cell exhaustion in Nectin-2 (CD112) KO mouse HCC model [44]. Alternatively, high expression of CD112 has also been observed in liver metastasis from colorectal cancer [72]. However, a study of 159 human subjects diagnosed with HCC demonstrated that the expression of CD112 in the cancer tissue specimens was lower than that in the surrounding peritumoral liver tissues [73]. Studies also strongly link decreased CD112 expression levels with increased levels of serum α-fetoprotein [74, 75]. Univariate Cox regression and Kaplan-Meier curve analyses have shown that decreased CD112 expression levels positively correlate with poor post-surgery OS [73]. Therefore, the correlation between CD112 expression and HCC prognosis may be dependent on extra- and inter-tumoral heterogeneity, and additional studies are required to confirm this relationship.

### Lung cancer

CD112 and nectin-4 appeared to be diagnostic in lung cancer; CD112 was reportedly a stronger diagnostic indicator, but neither biomarker was prognostic or predictive [76]. The authors found that serum concentrations of CD112 (5.0 ng/mL vs. 0.650 ng/mL; *p* < 0.0001) and nectin-4 (2.45 ng/mL vs. 1.15 ng/mL; *p* < 0.001) were significantly up-regulated in the cancer patients compared to those in the controls. Serum CD112 had higher sensitivity (91.9%) and specificity (92.5%) than serum nectin-4 (sensitivity, 70%; specificity, 85%), or a combination of serum CD112 and nectin-4 (sensitivity, 80%; specificity, 87%). Thus, the serum concentration of CD112 was more effective in identifying individuals with and without lung cancer than serum nectin-4 (alone or in combination with CD112). However, CD112 and nectin-4 levels in serum were not associated with the clinicopathological parameters, OS, or PFS. This study only assessed the serum concentration of CD112 in lung cancer patients. At present, CD112 expression in lung cancer tissues and adjacent tissues remains unclear, and its value as a prognostic marker needs to be investigated.

### Acute myeloid leukemia

In one study, CD155 and CD112 were reportedly negative prognostic markers of acute myeloid leukemia (AML) [77]. The study examined 429 AML patients, including 139 patients (cohort A) enrolled in the AMLSG 07–04 study of the German-Austrian Study Group (NCT00151242) [78] and 290 patients (cohort B) from the GEO database (GEO accession GSE6891) [79]. In cohort A, 94 and 95% of the patients expressed CD112 and CD155, respectively, and CD112 expression significantly correlated with the expression of CD155. Multivariate Cox proportional hazards model analysis indicated that a high CD112 expression level was correlated with a lower RFS (*p* = 0.017) and had a borderline significant negative impact on the OS (*p* = 0.087). In cohort B, no significant difference in CD112 expression occurred among various French-American-British subtypes. Furthermore, a Kaplan-Meier survival analysis confirmed that the OS of patients with high expression of CD155 and CD112 seems to be significantly lower than that of the low expression group. In sum, CD155 and CD112 expression had a significant impact on the prognosis of AML.

### Colorectal cancer

In one study [80], CD112 expression in the serum had a diagnostic value for colorectal cancer (CRC) patients; it appeared to have a poor prognostic value among the non-metastatic patients. The serum expression levels of CD112 in the CRC patients (non-metastatic and metastatic) were significantly higher than those in the healthy controls. Elevated concentrations of CD112 had a significantly adverse effect on the PFS compared to lower levels (median 5.8 vs. 9.1 months, respectively; *p* = 0.04). Although elevated serum levels of CD112 showed a significantly unfavorable effect on PFS in the non-metastatic patients (median 6.0 vs. 14.0 months, respectively; *p* = 0.05), there were no such effects in the metastatic patients. CD112 serum levels had no significant adverse effect on OS in all patients (*p* = 0.14). Furthermore, the serum levels of CD112 did not appear to have any significant effect on OS in the non-metastatic and metastatic group of patients (*p* = 0.32 and *p* = 0.07, respectively). However, high expression of CD112 could be found in metastatic colorectal cancer cells as discussed previously [72]. The role of CD112 involved in colorectal cancer metastasis needs to be further elucidated.

### Ovarian cancer

In a cohort of 60 ovarian cancer patients, the results showed that the high expression of CD112 was associated with lymph node metastasis and residual tumor after surgery [81]. Although, there were no significant differences between the histological subsets, tumor grades, receptor status, or metastasis [81], CD112 was overexpressed in ovarian cancer tissues and a variety of human ovarian cancer cell lines [82]. Anti-Nectin-2 mAbs have been shown to have an antitumor efficacy in vitro or mouse therapeutic models via strong ADCC [82]. Furthermore, patients with lymph node metastasis or residual tumor after surgery (size, < 1 cm) had higher gene expression levels of CD112 than those with a negative node or complete tumor resection [81]. Therefore, CD112 expression in ovarian cancer may support tumor cell adhesion and contribute to tumor growth and lymph node metastasis. The CD112 expression level decreased significantly in the peritoneal endothelial cells of the tumor patients compared to that in the healthy controls; alternatively, the expression level of vascular endothelial growth factor (VEGF) increased significantly in the serum of the tumor patients [81]. Also, CD112 levels were significantly down-regulated in endothelial cells stimulated by VEGF and increased significantly after the addition of VEGF inhibitors [81]. Therefore, CD112 down-regulation in the peritoneal vasculature might be driven by VEGF. This down-regulation increases vascular permeability and causes ascites, which could further mediate tumor spread in the abdominal cavity.

### Pancreatic ductal adenocarcinomas

In a cohort of 106 pancreatic ductal adenocarcinomas (PDAC) [83], high CD112 and DDX3 (DEAD-box helicase 3 X-linked) expression levels were associated with poor prognosis and progression. Positive CD112 and DDX3 expression appeared in 58 and 55 PDAC patients, respectively, but not in the 13 healthy pancreatic tissues. The expression rates of CD112 and DDX3 in PDAC tumor tissues were significantly higher than those in the peri-carcinomatous tissues, benign lesions, and healthy pancreatic tissues. Patients who presented with poor differentiation, invasion of the surrounding tissues and organs, lymph node metastasis, and TNM stages III and IV diseases had higher levels of CD112 and DDX3 than those with a well-differentiated tumor, no invasion, no lymph node metastasis, and TNM stages I and II disease. Kaplan-Meier survival and Cox multivariate analyses showed that patients with positive CD112 and DDX3 expression were more likely to have shorter survival than those with negative CD112 and DDX3 expression, and both CD112 and DDX3 were independent prognostic factors.

However, different conclusions emerged in another study on the prognosis of PDAC [84]. CD112 expression was not associated with OS, but the higher CD112’s immunohistochemistry score and the worse the histological grade. They also found that CD112 was weakly expressed in the membranes and cytoplasm of the adenocarcinoma cells and apical membranes of the intercalated ducts, intralobular ducts, and interlobular ducts in a healthy pancreas. Thus, the prognostic value of CD112 in PDAC needs to be further evaluated.

In summary, high CD112 expression was associated with aggressiveness and poor prognosis in gallbladder cancer, AML, and ovarian cancer. Serum CD112 levels have a diagnostic value in lung cancer and CRC, and high levels correlate with poor prognosis in CRC. However, its effect on the prognosis of PDAC and HCC remains to be further explored.

## Conclusion

Cancer immunotherapy studies have reached a significant milestone following the recent discovery of ICIs. However, a significant section of cancer patients has not benefited from these new immune checkpoint inhibitor pathways due to underlying primary and secondary drug resistance. The data reviewed in this study demonstrates the need for further research into alternative cancer therapies that can prevent the CD112R-CD112 interaction or act in combination with the TIGIT-CD155/CD112 blockade. Different ICIs and their pathways may show different functional profiles in a variety of tumor microenvironments. Thus, it is essential to accurately map the expression profile of each pathway in different types of tumors to determine the most suitable treatment that can guarantee optimal results.

## Data Availability

Not applicable.

## Abbreviations

ICIs: immune checkpoint inhibitors
CTLA-4: cytotoxic T lymphocyte-associated antigen-4
PD-1: programmed cell death protein-1
RCC: renal cell carcinoma
NSCLC: non-small-cell lung cancer
TME: tumor microenvironment
TIGIT: T cell immunoglobulin and ITIM domain
PVR: poliovirus receptor
NK: Natural Killer
DCs: Dendritic Cells
TILs: Tumor-infiltrating lymphocytes
CD112: cluster of differentiation 112
PVRL2: PVR-related protein 2
TLR: Toll-like receptors
TAMs: Tumor-associated Macrophages
NFAT: nuclear factor of activated T cells
IFN-γ: interferon-γ
LAG-3: lymphocyte activation gene-3
Tim-3: mucin domain-containing molecule-3
PFS: progression-free survival
OS: Overall survival
SC/ASC: squamous cell/adenosquamous carcinoma
AC: adenocarcinomas
AML: Acute Myeloid Leukemia
CRC: colorectal cancer
VEGF: vascular endothelial growth factor
PDAC: pancreatic ductal adenocarcinomas
DDX3: DEAD-box helicase 3 X-linked
